# Differential Characterization of Midgut Microbiota Between Bt-Resistant and Bt-Susceptible Populations of *Ostrinia furnacalis*

**DOI:** 10.3390/insects16050532

**Published:** 2025-05-18

**Authors:** Juntao Zhang, Ziwen Zhou, Xiaobei Liu, Yongjun Zhang, Tiantao Zhang

**Affiliations:** State Key Laboratory for Biology of Plant Diseases and Insect Pests, Institute of Plant Protection, Chinese Academy of Agricultural Sciences, Beijing 100193, China

**Keywords:** *Ostrinia furnacalis*, midgut bacteria, *Bacillus thuringiensis*, Cry toxin

## Abstract

*Bacillus thuringiensis* (Bt) is an efficacious biocontrol bacterium known for producing various toxins, such as crystal toxins, which disrupt the midgut epithelium of pest larvae, leading to larval mortality. However, the development of resistance to *Bacillus thuringiensis* in pests poses a significant threat to the widespread application of Bt corn. Consequently, we employed high-throughput sequencing of the midgut bacterial 16S ribosomal RNA to characterize the midgut bacteria in four Bt-resistant strains. Specifically, Bt-resistant strains (ACB-FR and ACB-AcR) exhibited lower bacterial diversity compared to ACB-AbR and ACB-IeR. Multivariate analyses and statistical evaluations further demonstrated that the microbiota communities in Bt-resistant pests (AbR, AcR, IeR, and FR) were distinct from those in Bt-susceptible strains. Notably, the genus *Klebsiella* predominated in BtS, whereas *Enterococcus* was the genus with peak enrichment in AbR, AcR, IeR, and FR. Bioassays subsequently revealed that *Enterococcus* enhances the Cry1Ab resistance of ACB larvae. Our investigations indicate that treatment with Bt protein alters the midgut microbiota community of *O. furnacalis*, and these microbiota differences may potentially modulate the Bt-induced lethality mechanism.

## Simple Summary

*Bacillus thuringiensis* (Bt) is extensively used all over the world as a type of eco-friendly pesticide. Despite its high efficacy against various pests, the main challenge is the emergence of pest resistance. In this study, we verified the differences of midgut microbiota in four Bt-resistant strains and certified that *Enterococcus* enhances the Cry1Ab resistance of the Asian corn borer by bioassays.

## 1. Introduction

*Bacillus thuringiensis* (Bt), is a ubiquitous Gram-positive, endospore-forming bacterium originally characterized as an entomopathogen due to exhibiting insecticidal activity primarily through crystalline (Cry) proteins produced during sporulation, which accumulate as parasporal inclusions [1]. These production are primarily composed of one or more proteins known as crystal (Cry) and cytolitic (Cyt) toxins, also referred to as δ-endotoxins [2]. These proteins (protoxins) are activated by insecticidal midgut proteases, and the activated toxins affect the larval midgut epithelium, causing a collapse of the membrane and ultimately leading to insect death [3]. Bt gene-modified crops have been globally adopted as a sustainable for agricultural pest control and human disease vector management. Nevertheless, the emergence of resistance in pests compromises the sustainable deployment of Bt-based crop protection strategies [4].

The Asian corn borer, *Ostrinia furnacalis* (Guenée), which is abbreviated as ACB, is one of the most economically significant pests of maize in China. Research on the Asian corn borer has been conducted since the 1930s [5]. The larvae exhibit broad feeding behavior across all maize tissues, of which the most severe crop damage results from their drilling activity in developing ears and stalks [6]. The Asian corn borer can cause significant maize losses, estimated at 6–9 million tons for an ordinary year. However, most maize fields are left without any effective resistance measures. Field tests in China have shown that maize expressing the Bt toxin (MON810) have significantly increased ACB larval mortality [7]. However, quantitative bioassays revealed that lab-selected ACB strains achieve resistance ratios (RR_50_) of >150 (Cry1Ab), 350 (Cry1Ac), >800 (Cry1Ie), and 1700 (Cry1F) [8,9,10,11]. This rapid evolution of resistance may severely limit the expanding application of genetically modified maize.

Laboratory and field data have identified three different resistant mechanisms in pests to Bt crops, namely, altered toxin activation, mutations in toxin-binding receptors, and the immune response system [12]. Comparing the toxin receptor sequences between Cry1Ab-resistant and -susceptible ACB colonies, the resistant colony decreased some or all of their binding capacity, presumably by altering one or more of its shared binding sites [13]. Research and laboratory experiments demonstrated that Cry1 proteins bind to several membrane-associated receptors in the midgut epithelium of *Lepidopteran* larvae, such as aminopeptidase N, cadherin, and ABC transporters [14,15,16,17]. Several studies provide evidence confirming that the r1–r3 alleles of the Lepidopterous larvae cadherin gene BtR confer recessively inherited resistance to the Cry1Ac toxin [18]. Nevertheless, the accurate mechanism underlying ACB resistance to Bt remains not fully proven. The insect gut contains many different kinds of microbiota that may contribute to insect hosts surviving and adapting to the environment [19,20,21]. Numerous studies have revealed that midgut bacteria can influence the pest resistance of Bt while potentially contributing to the insect Bt resistance development [22]. Mortality caused by Cry1 toxin exposure in species such as *Vanessa cardui, Manduca sexta,* and *Pieris rapae* was reduced under an antibiotics diet to decrease the gut microbiota. However, reintroducing the native *Enterobacter* sp. restored high toxicity [23]. Bt toxin susceptibility was also found to decrease in *Plodia interpunctella* after removing the gut bacteria [24]. These experiments demonstrate that gut bacteria critically modulate Bt toxin efficacy to pests, especially in Lepidoptera.

To investigate potential associations between midgut microbiota and Bt resistance in ACB, we compared the microbial communities of five ACB strains (BtS, AbR, AcR, IeR, and FR) using 16S rRNA Illumina sequencing. Our study investigated how gut microbiota modulate Bt resistance and susceptibility in the Asian corn borer with a focus on characterizing functional differences and interactions between resistant and susceptible host strains.

## 2. Materials and Methods

### 2.1. Mass-Rearing and Artifical Selection of Bt-Resistant ACB Strains

Five laboratory strains of ACB were used in this research, namely, a Bt-susceptible strain (S) and four laboratory resistant strains (BtR) selected under different Cry toxins: Cry1Ab (AbR), Cry1Ac (AcR), Cry1F (FR), and Cry1Ie (IeR).

All colonies were obtained from Institute of Plant Protection, Chinese Academy of Agriculture Sciences. The susceptible strain (S) was maintained on an artificial diet [25] under Bt toxin-free conditions to preserve baseline susceptibility. Resistance levels to Bt toxins were determined through 7-day diet-incorporated dose–response bioassays for AbR, AcR, FR, and IeR strains, respectively. The AbR, AcR, FR, and IeR strains have respectively tested more than 710-, 400-, 500-, and 800-fold resistance ratios under bioassays. All colonies were reared in a controlled climate chamber maintained at 27 ± 1 °C, 70–80% relative humidity, under a 16:8 h (light/dark) photoperiod.

### 2.2. Dissection of Gut Tissues and Extraction of DNA

The fifth instar ACB larvae were surfaced-sterilized by immersion in 70% ethanol for 3 min, followed by three saline washes (5 s each) to remove residual ethanol. The larvae were first immobilized via cold anesthesia (5 min on crushed ice) before dissection in a laminar flow hood. Midgut tissues were harvested using sterilized microsurgical tools in sterile saline solution and immediately flash-frozen in liquid nitrogen and stored at −80 °C until genomic DNA extraction. Each pooled sample was collected from the midguts of fifty-fifth-instar larvae representing different strains. Genomic DNA was extracted from dissected midgut tissues using a TGuide S96 Magnetic Soil/Stool DNA Kit (Tiangen Biotech Beijing Co., Ltd., Beijing, China) in accordance with the manufacturer’s protocol. DNA concentrated samples were quantified using the Qubit dsDNA HS Assay Kit and Qubit 4.0 Fluorometer (Thermo Fisher Scientific, Eugene, OR, USA) [26], and DNA integrity and fragment size distribution were evaluated by electrophoresis on a 1% (*w*/*v*) agarose gel stained with GeneGreen Nucleic Acid Dye (Tiangen Biotech Beijing Co., Ltd., Beijing, China), with visualization under UV light.

### 2.3. Amplification of 16S rRNA Gene Sequences for Microbial Community

The V3–V4 region of 16S rRNA gene from the ACB midgut microbial community was amplified using universal primers (338F: 5′-ACTCCTACGGGAGGCAGCA; and 806R: 5′-GGACTACNNGGGTATCTAAT). PCR amplifications were set up in a 10 µL reaction system that contained a 10 ng template, 0.2 μM of each primer, 1× loading buffer, and nuclease-free water. Target bands were purified using AgencourtAMPure XP Beads (Beckman Coulter, Indianapolis, IN, USA) and quantified using the Qubit dsDNA HS Assay Kit on a Qubit 4.0 Fluorometer (Invitrogen, Thermo Fisher Scientific, Eugene, OR, USA). Libraries were prepared and sequenced on an Illumina NovaSeq 6000 platform (Illumina, Santiago, CA, USA), generating 250 bp paired-end reads [26].

### 2.4. PCR-Amplified DNA Sequencing

Following total DNA extraction from all the midgut tissues, specific primers were designed based on conserved region sequences. Illumina-compatible sequencing adapters were incorporated into the primers tails to enable downstream library preparation. The Amplification PCR products underwent purification, quantified via fluorometry (Qubit 4.0), and normalized to equimolar concentrations (10 nM) for pooled library construction. The qualified libraries were sequenced with Illumina HiSeq 2500. Raw image data files were converted into sequence reads through base calling. The optimized sequences were filtered and pair-ended merged to obtain optimized sequences (Tags). The optimized sequences were clustered and assigned to operational taxonomic units (OTUs), and species classification was performed according to the sequence composition of each OTU [27]. The number of sequences was statistically processed to assess the quality of the data. The tags were clustered at 97% similarity level using UCLUST in QIIME (version 2.0.0) software to identify OTUs [28]. OTUs were annotated based on the Silva (bacteria) and UNITE (fungal) taxonomic databases.

### 2.5. Midgut Bacteria Diversity Analysis

The taxonomic information of each OTU was obtained by comparing the representative sequences of OTUs with the microbial reference database. This enabled the quantitative analysis of microbial community composition across taxonomic levels (phylum, class, order, family, genus, and species) for each sample. Using QIIME software, species abundance tables were generated at different levels, and community structure maps were created at different taxonomic levels using the R language tool.

### 2.6. Isolation and Characterization of Enterococcus and Klebsiella Species

The genera *Enterococcus* and *Klebsiella* were found to be highly dominant in Bt-resistant strains. To isolate these bacterial genera, 5th-instar larvae of ACB were first immersed in 70% ethanol for 3 min to eliminate surface bacteria. Subsequently, the midgut was dissected under sterile conditions and collected in sterile centrifuge tubes containing 20 μL of sterile water. The gut contents were then homogenized using sterile grinding pestles. The resulting liquid was streaked onto MIAC medium plates (Qingdao Hi-Tech Industrial Park Hope Bio-Technology Co., Ltd., Qingdao, China), which are specifically designed for the cultivation of *Klebsiella* species and cultured at 30 °C for 24 h. For the isolation of *Enterococcus* species, the gut liquid was streaked onto bile aesculin azide agar plates (Qingdao Hi-Tech Industrial Park Hope Bio-Technology Co., Ltd., Qingdao, China). The experiment was conducted with three biological replicates. Bacteria exhibiting identical morphologies were selected for subculture on the corresponding medium plates.

For the identification of *Enterococcus* species, 10 bacterial colonies were chosen for PCR amplification using the 16S rRNA gene with universal primers Ent-27F (5′-AGAGTTTGATCCTGGCTCAG-3′) and Ent-1492R (5′-TACGGTTACCTTGTTACGACTT-3′). Similarly, for the identification of *Klebsiella* species, 10 bacterial colonies were selected and amplified with specific primers Kle-F: TGGCCCGCGCCCAGGGTTCGAAA and Kel-R: GATGTCGTCATCGTTGATGCCGAG. PCR products were characterized by Sangon Biotech Co., Ltd. (Shanghai, China) and then subjected to BLAST (version 2.14.0) searches against the National Center for Biotechnology Information (NCBI) database for identification. Phylogenetic analysis was performed using the neighbor-joining method in MEGA 11 software.

### 2.7. Virulence Assay of E. faecalis and Cry1Ab Susceptibility After Levofloxacin Treatment

The virulence of *E. faecalis* on ACB larvae was assessed using feeding and injection methods. For the feeding assay, 50 mL of *E. faecalis* solution in logarithmic growth stage was centrifuged at 4000 rpm for 10 min to discard supernatant. Then, we blended it with LB liquid medium to OD600 = 1.0 solution and incorporated it into an artificial diet with five bacteria concentration gradients, which with a normal diet served as the control. Each treatment included 24 larvae and was repeated three times. For the injection assay, enterococcal solution at the logarithmic growth stage was centrifuged at 3500 rpm for 15 min, and the supernatant was discarded. Then, we blended it with deionized water to OD600 = 1.0 solution, which was injected 1 μL into 3 instar ACB larval hemolymph while injecting deionized water as the control group.

The role of *E. faecalis* in Cry1Ab resistance was investigated by supplementing artificial diet with levofloxacin (1 mg/mL). After feeding ACB larvae with the levofloxacin-supplemented diet for 24 h, the larvae were divided into two groups: one group was transferred to an artificial diet containing Cry1Ab protein (LC50 = 6.28 ng/cm^2^), and the other was transferred to a diet containing both Cry1Ab protein and *E. faecalis*. Treatment groups (*n* = 24 larvae each) were independently replicated in triplicate.

GraphPad Prism 9.5.0 was used to statistically analyze the results of virulence of *E. faecalis* and Cry1Ab protein virulence bioassay on ACB larvae, while the *t*-test and one-way ANOVA were used to determine the statistical significance.

## 3. Results

### 3.1. Overview of the 16s-RNA Sequencing Data

The V3-V4 region of 16S rRNA was amplified and sequenced from five samples, generating 438,890–746,708 raw reads. After quality control and chimeric removal, 391,221 to 664,608 high-quality tags were retained (Table 1). The sequences clustered into 1640–2754 OTUs per sample at 97% similarity.

### 3.2. Different Gut Bacterial Communities in BtS and BtR ACB Strains

#### 3.2.1. The Relative Abundance of Species at the Genus Level

**Figure 1:**
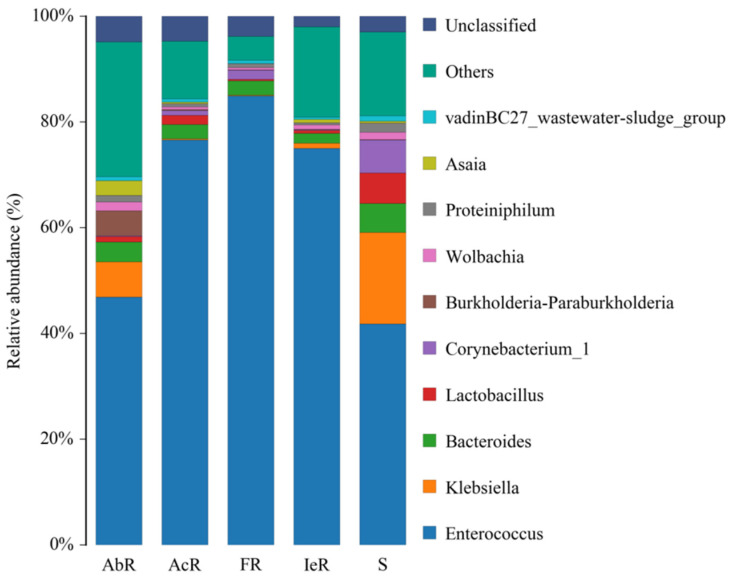
Relative abundance of the top 12 of midgut bacteria genera from BtR (AbR, AcR, FR, and IeR) and BtS (S) strains of Asian corn borer.

Dominant bacterial genera across the five strains were *Enterococcus* (41–83%), *Klebsiella* (0.1–19%), and *Bacteroides* (3–12%) (Figure 1), while *Enterococcus* exhibited the highest relative abundance in Bt-resistant strains.

The *Enterococcus* abundance in ACB resistant strains (45% for AbR, 73% for AcR, 83% for FR, and 70% for IeR), as well as the susceptible strain (41% for S) were compared. The relative abundance of *Lactobacillus*, *Klebsiella,* and *Bacteroides* were significantly higher in Bt-susceptible strains than in Bt-resistant strains.

#### 3.2.2. Isolation and Identification of Microorganisms

**Figure 2:**
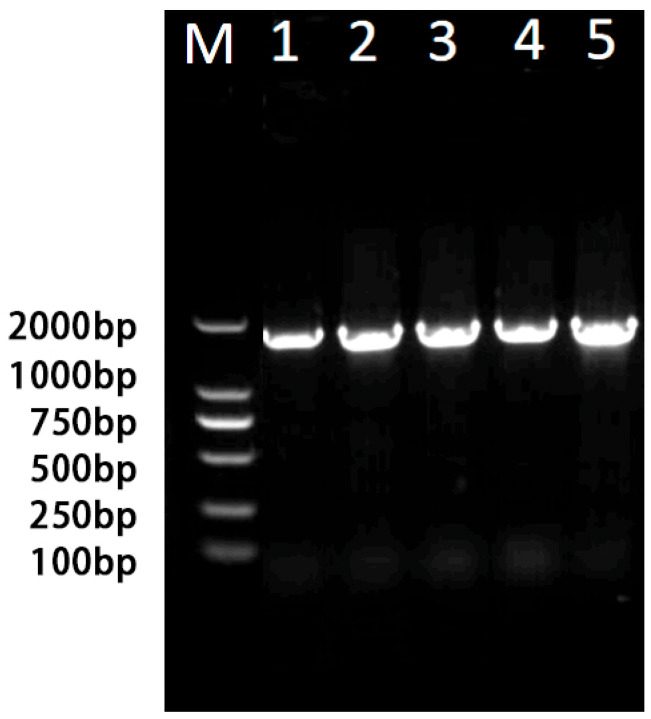
Agarose gel electrophoresis of *Enterococcus*. M: 2000 bp marker. 1–4: Bacterial solution electrophoresis results. 5: Positive control.

PCR amplification was performed on 10 randomly selected bacterial colonies, successfully identifying *Klebsiella* and *Enterococcus* species. The target fragment size for *Klebsiella* was approximately 300 bp, while for *Enterococcus* was approximately 1300 bp. We added *Enterococcus* spp. as positive control. The agarose gel electrophoresis results indicate that the selected bacterial isolate belongs to the *Enterococcus* genus (Figure 2). Phylogenetic analysis using MEGA 11 software identified the isolated the *Enterococcus* strain as *Enterococcus faecalis*.

### 3.3. The Impact of Gut Microbiota on Cry1Ab Resistance in Asian Corn Borer

#### 3.3.1. Virulence Evaluation of *Enterococcus faecalis* on ACB Larvae

**Figure 3:**
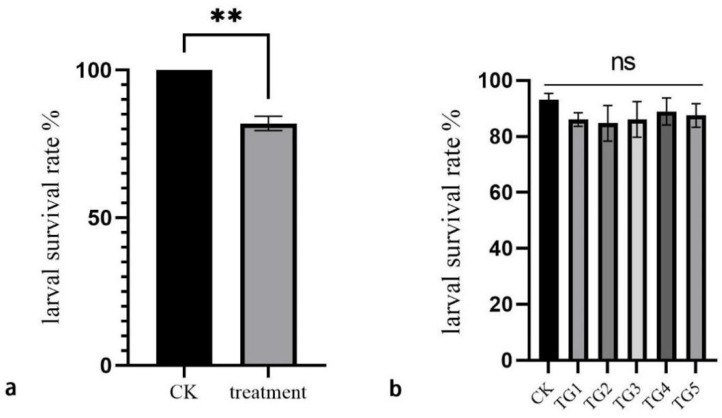
Larval survival rate of Asian corn borer after injection or feeding with *E. faecalis.* (**a**) Asian corn borer larval survival rate after injecting double-distilled water (CK) and *E. faecalis* (treatment). (**b**) Asian corn borer larval survival rate after feeding on normal diet (CK) and *E. faecalis* diet 0.0625 mg/mL (TG1), 0.125 mg/mL (TG2), 0.25 mg/mL (TG3), 0.5 mg/mL (TG4), and 1 mg/mL (TG5). “**”: indicates the difference is significant between CK and treatment at 0.01 level; ns: indicates no significant between CK and different treatments.

The results indicated that ACB larvae fed on diets with different *Enterococcus* concentration gradients maintained survival rates above 80%, with no significant differences compared to the control diet. However, larvae injected with the *Enterococcus* solution exhibited significantly reduced survival rates (Figure 3).

#### 3.3.2. *E. faecalis* Influence in ACB of Cry1Ab Resistance

**Figure 4:**
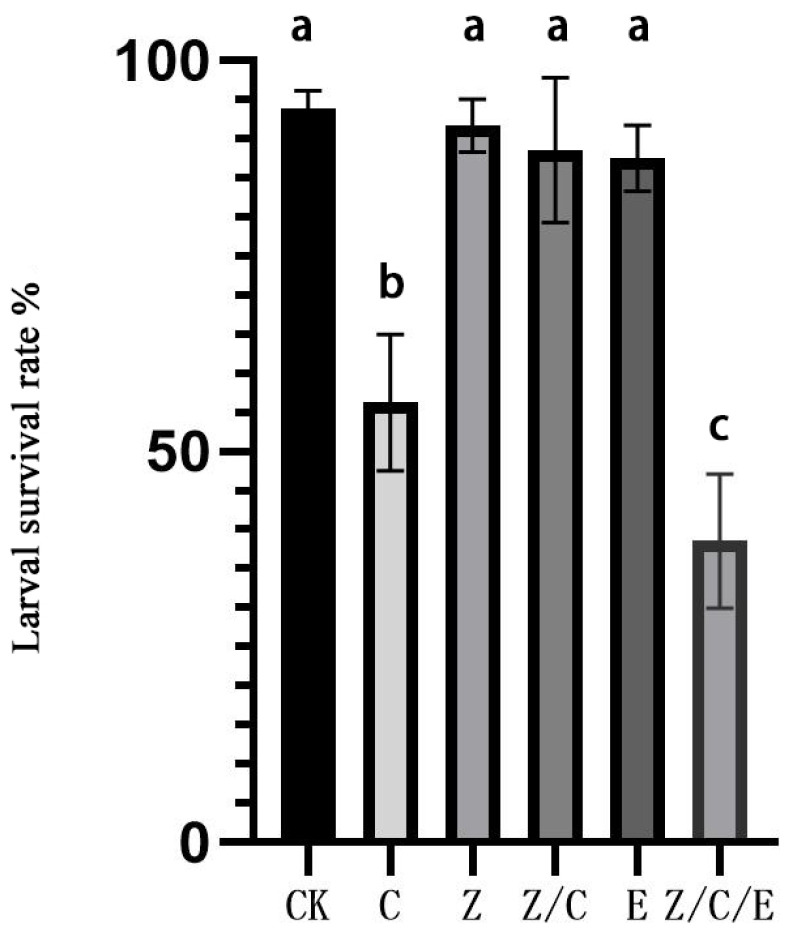
Survival rate of ACB larvae feeding on different treatments with artificial diet. CK: normal artificial diet; C: normal artificial diet containing Cry1Ab protein; Z: normal artificial diet with levofloxacin-supplemented; E. normal artificial diet containing *E. faecalis*; Z/C: normal artificial diet containing Cry1Ab protein and levofloxacin; Z/C/E: normal artificial diet containing Cry1Ab protein, levofloxacin, and *E. faecalis*. Different low case letters above columns indicate statistical differences at *p* < 0.01.

The experiment yielded several noteworthy findings. The survival rate of newly hatched larvae continuously fed with levofloxacin was 91.67%, compared to 93.75% in the control group fed with a regular diet, showing no significant difference between the two groups. However, the survival rate of larvae treated with levofloxacin for 24 h and then fed on an artificial diet containing Cry1Ab protein (at a concentration of LC50 = 6.28 ng/cm^2^) was significantly higher than that of larvae directly fed on the Cry1Ab-containing artificial diet. When larvae treated with levofloxacin were fed on a diet containing both Cry1Ab protein and *E. faecalis*, their survival rate was 38.54%, which was significantly different from the group Z/C serving as the control group (*p* < 0.001) (Figure 4).

## 4. Discussion

Different *Bacillus thuringiensis* products have been developed for pest control in agriculture and also against mosquito species [29]. Transgenic Bt plants have been proven effective for controlling Lepidopteran pests. However, since the first Bt resistance news of *Plodia interpunctella* occurred in 1985 [30], many more similar cases were reported. These findings raise concerns about the long-term efficacy of Bt toxins and pose a significant challenge to maintaining the effectiveness of both Bt-based pesticides and gene-modified plants expressing Bt toxins. *Plutella xylostella* was the only insect to eventually develop resistance to Bt applied as a biopesticide in previous research [31]. However, laboratory selection experiments revealed that over 50% of tested moth species (Noctuidae, Plutellidae, and Pyralidae) developed >10-fold resistance to Bt toxins, underscoring the widespread evolutionary capacity of Lepidoptera to adapt to Bt pressure [32]. This highlights an urgent need to decipher resistance mechanisms beyond traditional explanations (e.g., receptor mutations), including the overlooked role of gut microbiota.

Recent evidence suggests that the insect midgut significantly modulates host susceptibility to Bt toxins. Dominant bacterial genera in Lepidopteran midguts, such as *Pseudomonas*, *Bacillus*, *Enterobacter*, and *Enterococcus*, are not only vital for nutrient assimilation but also promote detoxification of plant secondary metabolites, potentially altering toxin efficacy [33,34]. After feeding gypsy moth larvae with different concentrations of antibiotics to reduce the midgut bacteria, the mortality of larvae fed Bt toxin was inversely proportional to the antibiotic concentration. This reduction in mortality was accompanied by reduced populations of culturable *Enterococcus* and *Enterobacter* from the midguts of larvae [22]. Bt protein treatment can alter gut microbial community composition in *Spodoptera exigua* compared with the normal populations [35]. Bt toxin exposure caused severe midgut epithelial disruption, which enables gut bacteria to translocate into the hemocoel [36]. This bacterial invasion may convert commensal bacteria into pathogens that potentially accelerate larvae mortality. In a previous study about *Manduca sexta*, Mason et al. [37] found that a common gut microbiota *E. faecalis* invades the hemolymph of *M. sexta* larvae within 48 h post-ingestion. The bacterial load increased progressively until death, though the precise translocation mechanisms remain unknown. This hypothesis was supported by the results presented in our study. Chen et al. [38] demonstrated that indigenous *Enterococcus* spp. synergistically enhanced Cry1Ca-mediated mortality in *Chilo suppressalis* larvae by inducing melanization and hemocyte apoptosis. Therefore, exploring the functional properties of *Enterococcus* in pest midguts could be a critical avenue for future research on of Cry-toxin resistance mechanisms.

In previous research, it was demonstrated that Bt proteins induce the collapse of the membrane, subsequently facilitating the invasion of midgut microbiota into the hemolymph and ultimately resulting in insect death due to sepsis [3]. In this study, ACB larvae fed on *E. faecalis* diet showed no significant difference in survival compared to those on a normal diet. However, direct midgut injection of *E. faecalis* substantially altered larval survival (Figure 3). Notably, when gut bacteria (including *E. faecalis*) were depleted using a levofloxacin-containing diet, ACB survival rates on Cry1Ab-treated feed significantly increased (Figure 4). These results demonstrate that *E. faecalis* enhances ACB susceptibility to Cry1Ab and suggest that gut microbiota composition critically modulates larval resistance to Bt toxins. This outcome aligns with the results of prior studies, which reported that a reduction in the gut microbiota of the diamondback moth leads to a decreased sensitivity of its larvae to the Cry1Ac protein [39]. Although the mechanism by which ACB reduces *E. faecalis* remains unknown, hemocytes from *Heliothis virescens* larvae exposed to Cry1Ac toxin exhibited upregulated expression of immune genes associated with Bt intoxication, including antimicrobial peptides, cytokines, and protease inhibitors [40]. We hypothesize that Cry1Ab-resistant ACB strains mount a similar immune response to suppress *E. faecalis* in the midgut.

In conclusion, this study reveals that the larval midgut bacteria participate in the death mechanism of Cry toxins in *O. furnacalis.* The microbial community composition and richness in Bt-resistant and Bt-susceptible strains were significantly different. We hypothesize that the midgut bacteria may influence Bt toxin resistance in many pests, though this requires experimental validation in future studies. The implications are significant for Bt-gene modified corn cultivation and may lead to new strategies for biological pest control.

## Figures and Tables

**Table 1:** Statistics of different ACB strains sequencing data.

Strain IDs	PE Reads	Raw Tags	Clean Tags	Effective Tags	AvgLen (bp)	GC (%)	Q20 (%)	Q30 (%)	Effective (%)
AbR	476,918	446,887	400,958	399,519	425	53.58	94.84	90.54	83.77
AcR	720,884	676,881	603,479	600,280	428	52.89	94.73	90.37	83.27
FR	793,527	746,708	666,106	664,608	429	52.77	94.79	90.51	83.75
IeR	696,613	654,749	586,431	583,344	427	53.10	94.82	90.52	83.74
S	467,691	438,890	393,593	391,221	426	53.23	94.89	90.62	83.65

## Data Availability

The original contributions presented in this study are included in the article. Further inquiries can be directed to the corresponding author.
